# Aging of hospital physicians in rural Japan: A longitudinal study based on national census data

**DOI:** 10.1371/journal.pone.0198317

**Published:** 2018-06-01

**Authors:** Masatoshi Matsumoto, Kazuki Kimura, Kazuo Inoue, Saori Kashima, Soichi Koike, Susumu Tazuma

**Affiliations:** 1 Department of Community-Based Medical System, Graduate School of Biomedical and Health Sciences, Hiroshima University, Hiroshima, Japan; 2 Department of Community Medicine, Chiba Medical Center, Teikyo University School of Medicine, Chiba, Japan; 3 Department of Public Health and Health Policy, Graduate School of Biomedical and Health Sciences, Hiroshima University, Hiroshima, Japan; 4 Division of Health Policy and Management, Center for Community Medicine, Jichi Medical University, Tochigi, Japan; 5 Department of General Internal Medicine, Hiroshima University Hospital, Hiroshima, Japan; Public Library of Science, UNITED KINGDOM

## Abstract

**Background:**

The disparity in the number of urban and rural physicians is a social problem in Japan. There may also be a disparity in the age of physicians. This study longitudinally examines both geographic and age distributions of physicians.

**Methods:**

Individual data from the Survey of Physicians, Dentists and Pharmacists in 1994, 2004 and 2014 and municipality data from the National Population Census were used. The 2015 municipality border was applied to all years, and all municipalities were classified into equal-size quintiles based on population density. Both municipalities and physicians were longitudinally observed.

**Results:**

Between 1994 and 2014, the number of physicians per 100,000 population increased by 31.8% in the most urban group of municipalities and 17.4% in the most rural group. The average age of physicians was highest in the most rural and lowest in the most urban group. The difference in average age between the urban and rural physicians widened from 2.1 years in 1994 to 6.0 years in 2014. This disparity is particularly pronounced among hospital physicians (from 1.5 years in 1994 to 7.6 years in 2014). In the most rural group, the number of hospital physicians younger than 40 years old has decreased by 59.4%, while the number of those 55–70 has grown by 153% and the number older than 70 years old by 41.0%. Between 1994 and 2004, only 23.0% of hospital physicians younger than 40 years old were retained in the most rural group; the retention rate fell to 19.3% between 2004 and 2014, while the rates increased in older physicians.

**Conclusions:**

The uneven distribution of physicians is increasing in Japan, as is the aging of rural hospital physicians. Shortage of physicians in rural areas may be more serious than that shown as their headcount.

## Introduction

The shortage of physicians in rural areas is a social problem in Japan as well as in other countries. Japanese national and prefectural governments have never had legal discretion over physicians’ practice locations, so physicians can choose where they work [1,2]. So despite the rapid increase in the number of physicians, their maldistribution has not changed [3,4]. University medical schools have traditionally been influential in pooling physicians and assigning them to urban and rural hospitals. However, the new residency training system for the physicians within the first two years after graduation was introduced nationwide in 2004 and these young physicians were allowed to choose non-university hospitals for their clinical training [5]. Since then, the conventional physician pooling and allocation system controlled by the university clinical departments has been weakened, exacerbating the maldistribution of physicians [6–8]. It is suspected that young physicians, are becoming more concentrated in urban areas [9]. Aging of physicians in rural Japan may be part of the the aging of Japan’s rural population where the percentage of elderly people is the highest in the world [10].

Both the number and the age distribution of physicians can be a matter of great concern. The aging of physicians in rural areas is a concern in some countries [11–13]. If the physicians are disproportionally older in rural than in urban areas, the maldistribution of physician workforce becomes even more serious than that shown as the headcount gap. The productivity of Japanese workers generally declines with age [14]. Among physicians, working hours also decrease with age increases [15]. The evidence outside Japan suggests that increasing age of physicians is associated with the decrease in their performance [16,17]. Thus from the perspective of labor force participation, the shortage of physicians in rural areas may be even worse than it appears, though older physicians have more years of clinical experience and the quality of their service might be better than younger ones.

Thus, to estimate and predict the workforce distribution of physicians in Japan as well as in other countries, we need to focus not only on the number but also on the difference in age between physicians in urban and rural areas. To date, no study has examined this age difference. The need for such a study is increasing in Japan where a new specialty training system for physicians after the two-year postgraduate residency training is scheduled to start in 2018. The new system has caused a great concern that young physicians will be even more likely to cluster in large cities [18–20]. This system was originally scheduled to start in 2017, but because of opposition from practicing physicians, policy makers and the population, the start was postponed [21].

In this study, we first examined the transition of physician distribution in Japan for the past 20 years. Then we compared the age composition of physicians in urban and rural areas, and examined whether this aging of physicians is more pronounced in rural areas. We also examined the movement of individual physicians, classified according to their ages, between urban and rural areas. Based on the results, we then discussed the likelihood of a physician shortage in rural Japan and its possible effects.

## Materials and methods

In this study we evaluated the geographic distribution of physicians based both in municipality-level aggregated data and individual-level follow-up data. The former is an ecological observation of municipalities and the latter is a longitudinal observation of each physician. Thus the study subjects are all municipalities and all physicians in Japan.

### Physician data

To analyze each physician’s practice location and age, we used individual data of the Survey of Physicians, Dentists and Pharmacists conducted in 1994, 2004, and 2014. Permission to use the data for this research was obtained from the Ministry of Health, Labour and Welfare. All licensed physicians in Japan are legally required to register for the Survey. For the purposes of the study, among all registered physicians, only those whose main employment was recorded as ‘practice’ in ‘clinics’ or ‘hospitals’ were recognized as ‘physicians’. A medical institution with 20 or more inpatient beds is defined by national law as a ‘hospital’ and that with fewer than 20 beds is defined as a ‘clinic’. The total number of physicians in each municipality (city, town, village, and special ward of Tokyo) was calculated from the Survey. Many municipalities were merged between 1994 and 2014 so their number has decreased substantially. The 2015 municipality border was thus applied to 1994, 2004 and 2014, and the number of municipalities was fixed in all years at 1729. To correct for this, we added the 2015 municipality code to 1994, 2004 and 2014 physician data using a table that indicates the correspondence between old codes and new ones. The correspondence table was created by Hibiya Computer System Co., Ltd (Tokyo, Japan). The number of physicians was then added up according to each new municipality code.

### Municipality data

Japan has three levels of government: municipal, prefectural and national. Data on municipal (city, town, village, and special ward of Tokyo) populations and land areas in 1995, 2005, and 2015, as the closest years to the Physician Census, was extracted from the National Population Census published by the Statistics Bureau, Ministry of Internal Affairs and Communications [22]. The 2015 municipality border was thus applied to 1995 and 2005 municipalities in the same way as physician data. The physician data was connected to this municipality-based population data through the municipality code.

### Classification of municipalities

All municipalities were classified into equal-size quintiles (from 1 to 5) according to the population density of each municipality in 2015. Quintile 1 was the group of municipalities with the highest population density (most urban), and quintile 5 was that with lowest (most rural). The cut-off value between quintiles 1 and 2 was 52.4, between 2 and 3 was 152.1, between 3 and 4 was 418.2, and between 4 and 5 was 1660.1 people per square kilometer. Population density, defined as the number of residents divided by land area including both habitable and non-habitable lands, is a well-established indicator of rurality-urbanity of an area [23]. In this study physicians who were in the same quintile of municipalities in the study period were regarded as being ‘retained’.

### Statistical analysis

The number of physicians per 100,000 population in each quintile of municipalities was calculated using the data of the total number of physicians and the total population. Because the years of physician data (1994, 2004, and 2014) are different from the years of population data (1995, 2005 and 2015), 1994 physician data was applied to 1995 population data, and 2004 physician data to 2005 population data, and 2014 physician data to 2015 population data. The physician data were gathered as of the end of December each year and population data were of the beginning of October, which means the real time gap between the two datasets was actually nine months.

The average age of physicians in each quintile of municipalities in each year was calculated. To analyze the age distribution of physicians, we classified physicians into five age groups: <40, 40–55, 55–70, and > = 70 years old. The number of physicians in each age group was calculated in each year both in the whole country and in each quintile of municipalities. All analyses distinguished hospital physicians from clinic physicians to compare the distribution of these two types.

To analyze the movement of individual hospital physicians, we merged the individual-level data in the 1994 Physician Census and that in the 2004 Physician Census using the physician identification number. Of the 151,299 hospital physicians in 1994, 127,925 (84.6%) could be merged with 2004 data, meaning that they were registered in both years of the Census. Of the 127,925 hospital physicians in 1994, 95,106 (74.3%) worked in hospitals in 2004. We also merged the data in the 2004 Physician Census and that in the 2014 Physician Census. Of the 163,683 hospital physicians in 2004, 140,120 (85.6%) could be merged with 2014 data. Of the 140,120 hospital physicians in 2004, 112,593 (80.4%) worked in hospitals in 2014.

Statistical analysis was done using SPSS version 24 (IBM-SPSS Japan, Tokyo). All maps shown in the Results were created using ArcGIS version 10.3.1 (ESRI Japan Inc.)

### Ethical consideration

This study was approved by the institutional review board of the Teikyo University School of Medicine (No. 17–038).

## Results

In this section, we first show the results of the municipality-level ecological analyses. Then we show the results of individual-level longitudinal analyses.

Table 1 shows the number of physicians and its change in each quintile of municipalities classified according to population density. The number of physicians nationwide has increased by 34.4% since 1994. The more urban the place, the greater the increase in its number of physicians. The increase was most remarkable in quintile 1 (most urban) municipalities (44.0%). In quintile 5 (most rural) municipalities the number of physicians has decreased (-10.8%). In other words, the skewed distribution of physicians toward urban areas has worsened.

**Table 1:** Changes in the number of physicians in municipalities, classified according to population density.

Municipalities		1994		2004		2014		Change 1994–2014	Change per year
Quintile	Population density*	n	%	n	%	n	%		
1 (most urban)	1660.1-	105761	47.9	125189	48.8	152311	51.3	44.0%	2.2%
2	418.2–1660.1	65013	29.4	75933	29.6	87267	29.4	34.2%	1.7%
3	152.1–418.2	33026	15	37275	14.5	40041	13.5	21.2%	1.1%
4	52.4–152.1	13713	6.2	14814	5.8	14272	4.8	4.1%	0.2%
5 (most rural)	-52.4	3310	1.5	3421	1.3	2954	1.0	-10.8%	-0.5%
Total		220823	100	256632	100	296845	100	34.4%	1.7%

* People per square kilometer

Between 1994 and 2014, Japan’s population grew by 1.2%. The population in the most urban quintile of municipalities rose by 9.3%, while that in the most rural quintile decreased by 24.0% (data not shown in illustrations).

**Fig 1:**
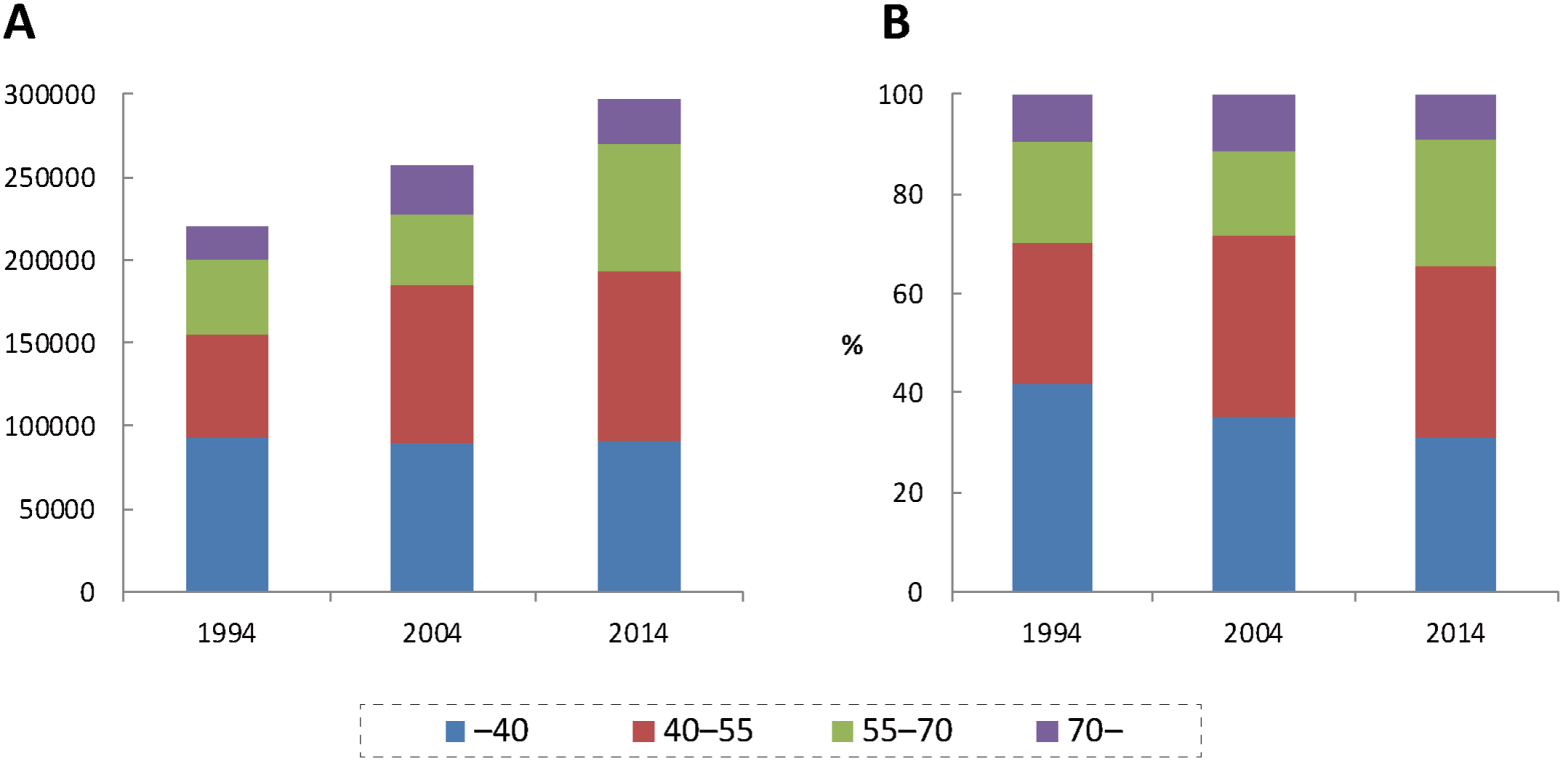
Age composition of physicians (A: Number; B: Proportion).

**Fig 2:**
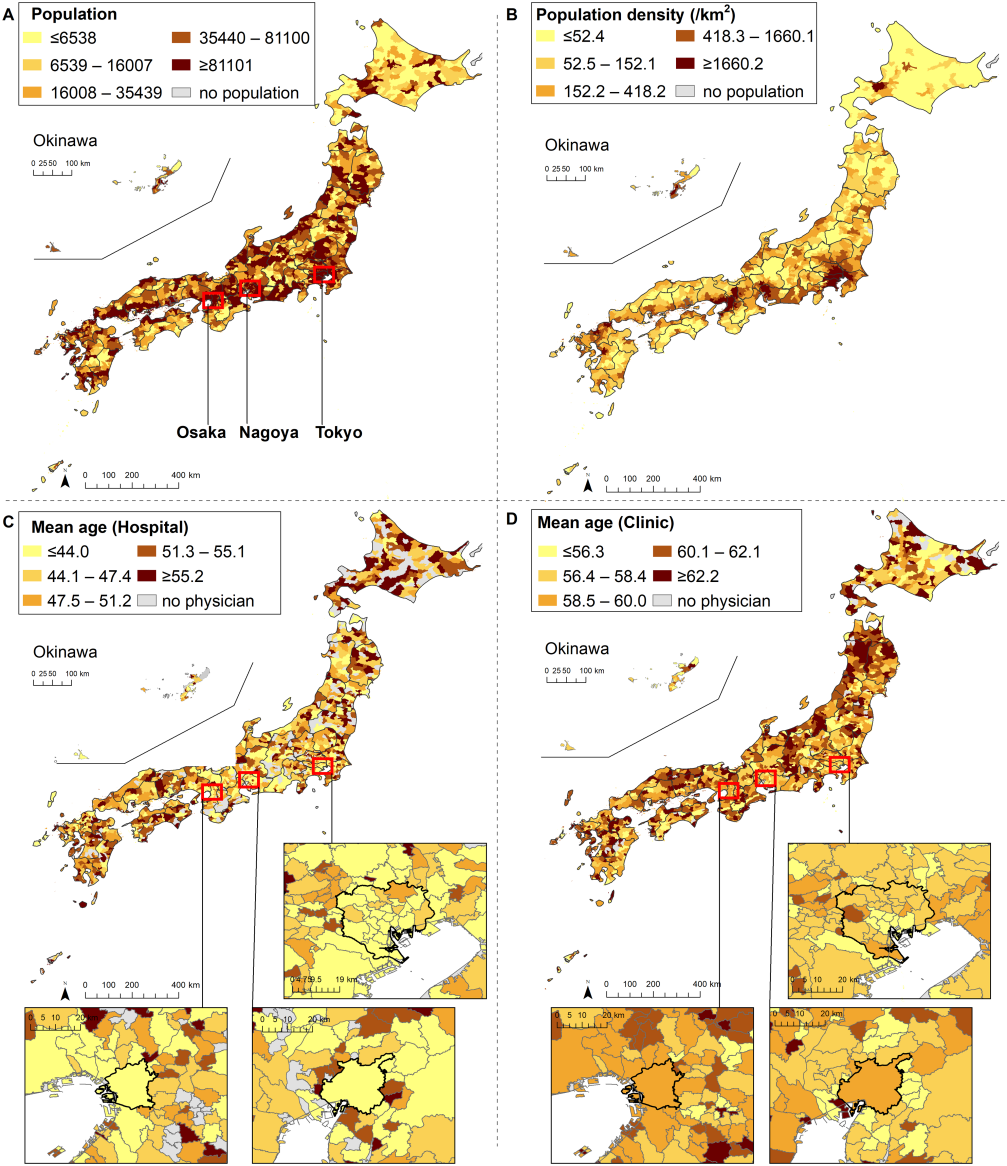
Population, population density and average age of physicians in each municipality in 2014.

The age composition of physicians in Japan is shown in Fig 1A (number) and 1B (proportion). Physicians are aging nationwide. The number of physicians who were 55–70 and >70 years old has increased substantially, while the number of those younger than 40 years old has not. The maps of municipalities colored according to population, population density and the average age of physicians in 2014 are shown in Fig 2A, 2B, 2C and 2D. Both the average age of hospital physicians and that of clinic physicians were higher in rural than in urban areas. As shown in S1 and S2 Figs, the decrease in the number of young physicians (<40 years old) is more remarkable in rural than in urban areas.

Spearman’s rank correlation coefficient between the mean age of physicians and log10-transformed population density of municipalities was -0.106 (n = 1,729, p<0.001) in 1994 and -0.239 (n = 1,729, p<0.001) in 2014 (data not shown in illustrations).

**Fig 3:**
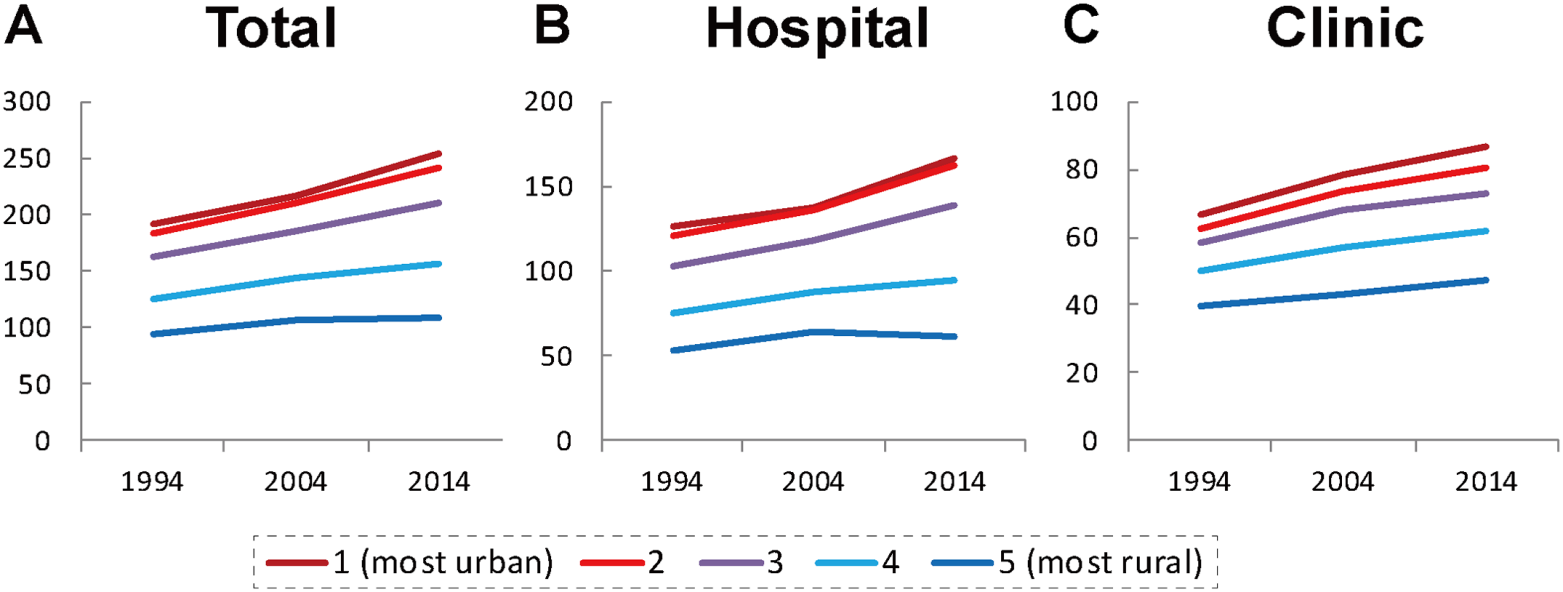
Number of physicians per 100,000 population in each quintile of municipalities (A: Total; B: Hospital; C: Clinic physicians).

The number of physicians per 100,000 population in each quintile of municipalities is shown in Fig 3A–3C. As for total physicians shown in Fig 3A, in quintile 1 (most urban), 2 and 3 municipalities, the physician-to-population ratios have steadily increased between 1994 and 2014 (31.8%, 32.1% and 30.4% respectively), while the increase rate in quintile 5 (most rural) municipalities was relatively small (17.4%). As shown in Fig 3B and 3C, the increase of hospital physician-to-population ratio in quintile 5 was slower (15.8%) than the increase in the clinic physician-to-population ratio in the same quintile (19.5%) between 1994 and 2014.

**Fig 4:**
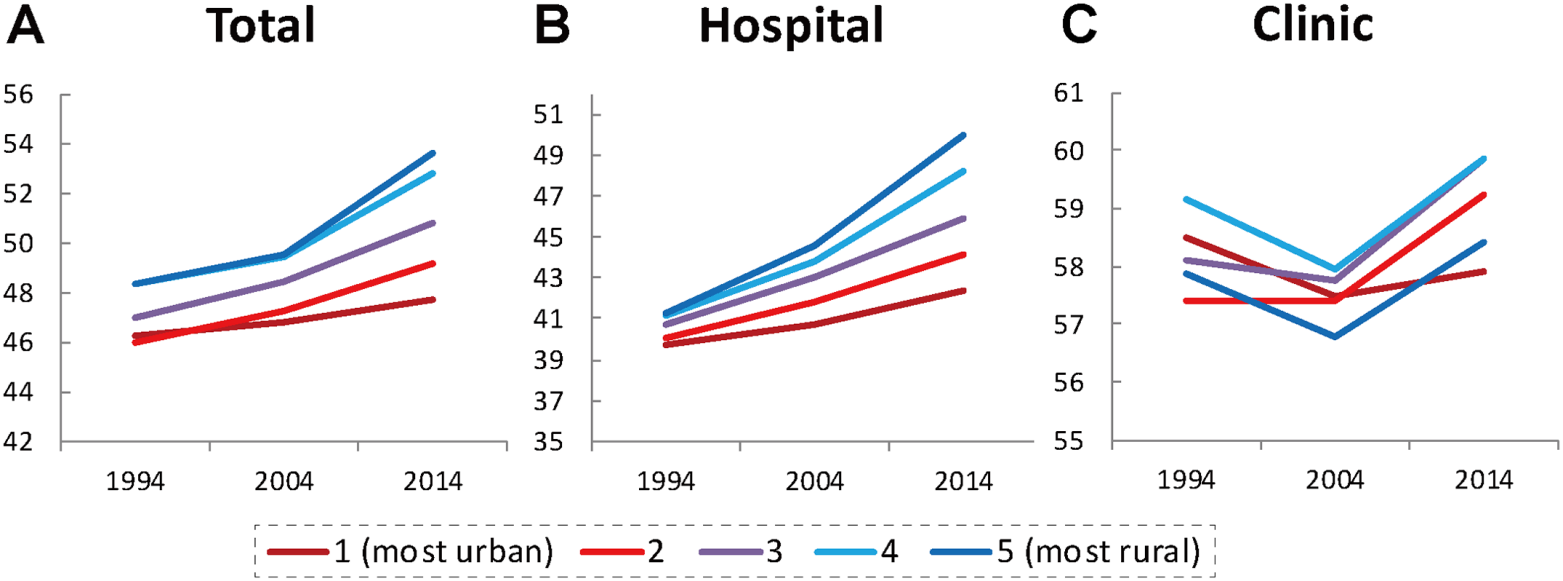
Average age of physicians in each quintile of municipalities (A: Total; B: Hospital; C: Clinic physicians).

The average age of physicians in each quintile of municipalities is shown in Fig 4. As shown in Fig 4A, the average age of total physicians was highest in quintile 5 (most rural) and lowest in quintile 1 (most urban) municipalities throughout the study period. In addition, the difference in average age between quintiles 1 and 5 has grown over the past 20 years (from 2.1 years in 1994 to 6.0 years in 2014). In other words, physicians in rural areas are older than those in urban areas and the gap has quickly widened. As shown in Fig 4B and 4C, the trend was more remarkable in hospital than in clinic physicians. The average age of hospital physicians in quintile 5 municipalities rose from 41.24 years old in 1994 to 49.94 years old in 2014. The gap in average age between quintiles 1 and 5 widened from 1.48 years in 1994 to 7.60 years in 2014. No similar trend has been observed in clinic physicians. The average age of clinic physicians in 2014 appears to be slightly higher than that in 1994, but there was a substantial decline in 2004, forming a v-shaped line.

**Fig 5:**
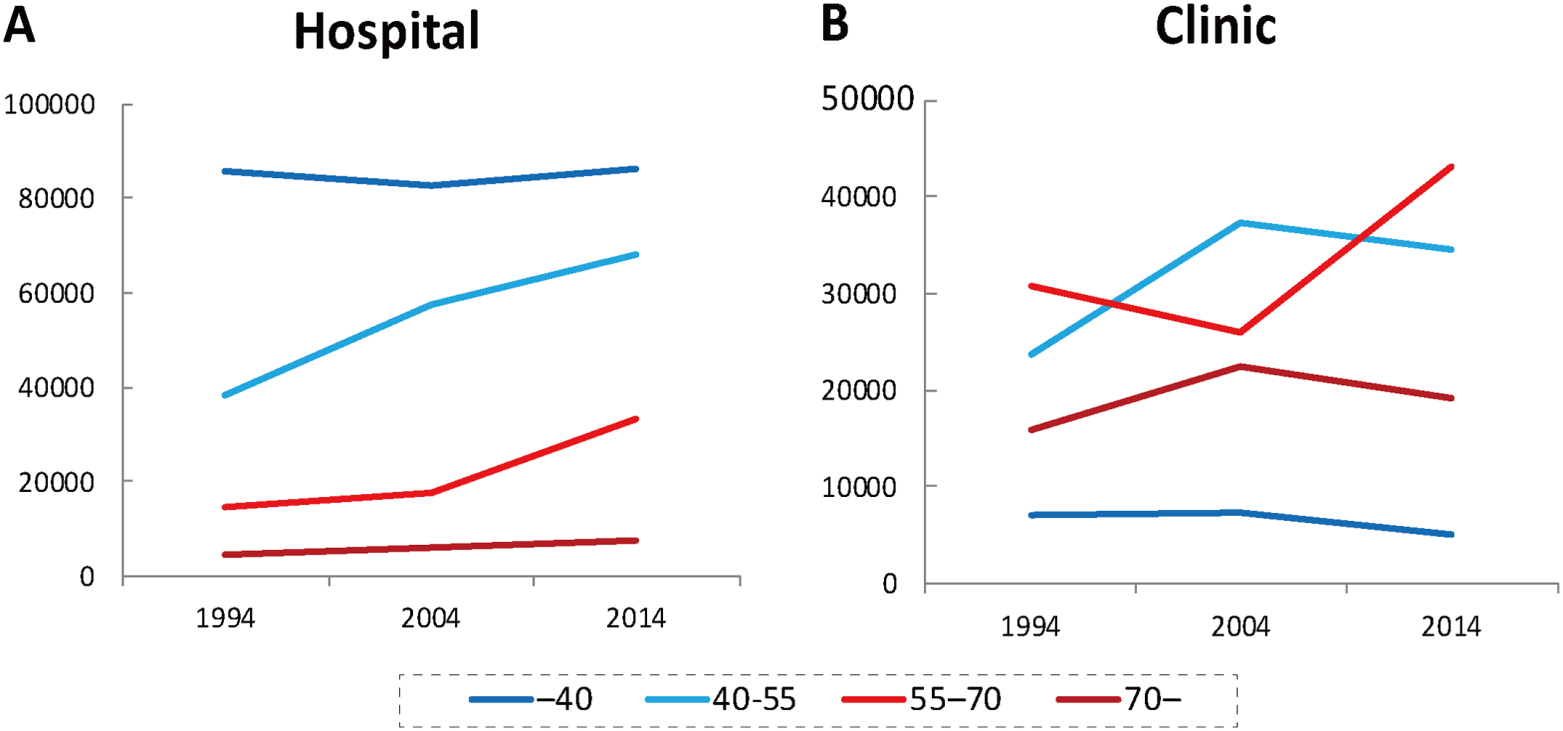
Age-specific number of hospital physicians (A) and clinic physicians (B).

The change in the age-specific number of physicians in each municipal category is shown in Fig 5A (hospital) and 5B (clinic). The number of hospital physicians between 40 and 55 years old has grown by 76.8% and that of those 55–70 grew by 124.9% between 1994 and 2014. At the same time, the number of hospital physicians who were younger than 40 years of age has increased only by 0.7%. This trend was not apparent among clinic physicians.

**Fig 6:**
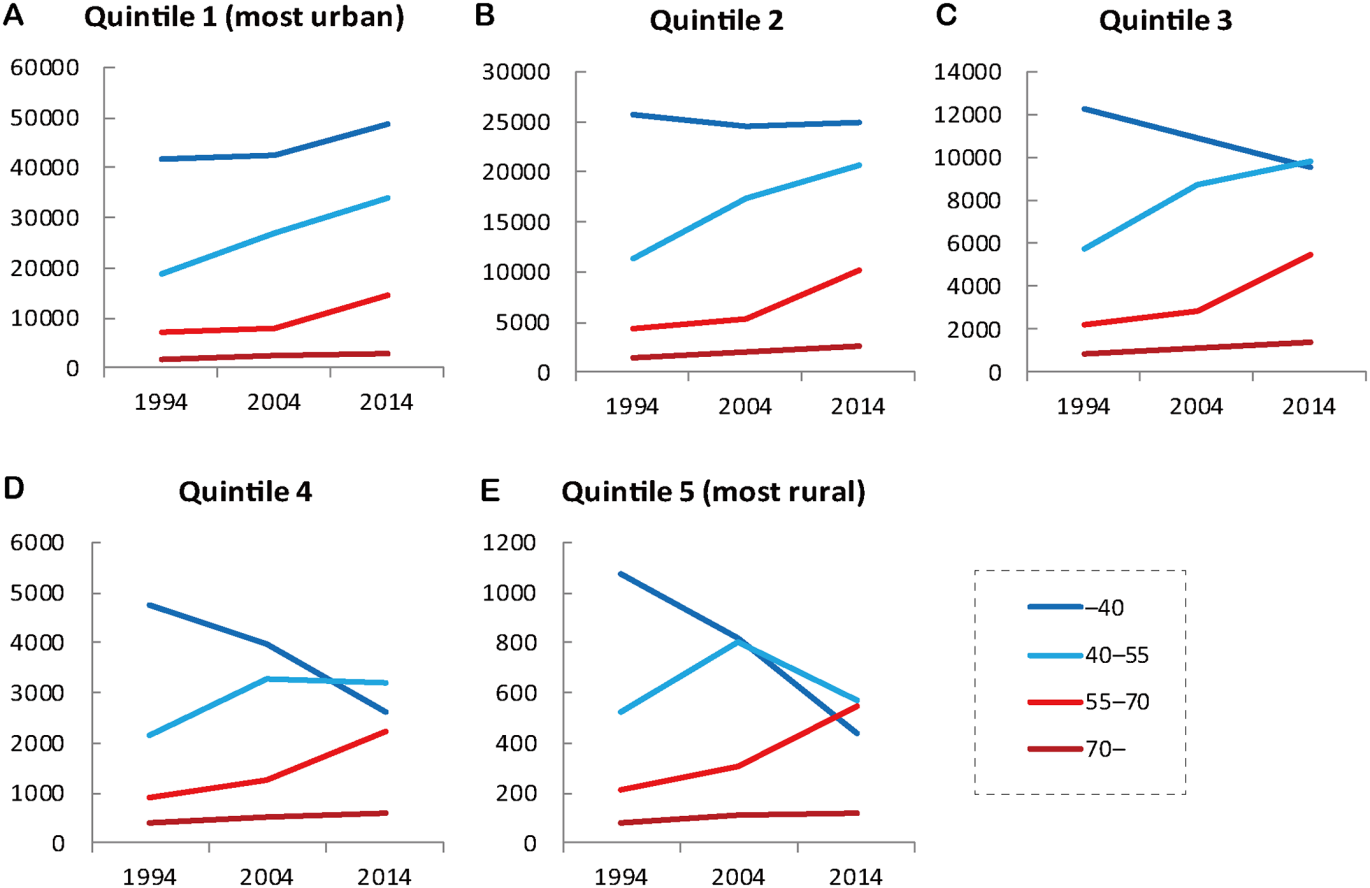
Age-specific number of hospital physicians in each quintile of municipalities.

The age-specific number of hospital physicians in each quintile of municipalities is shown in Fig 6A: quintile 1; 6B: quintile 2; 6C: quintile 3; 6D: quintile 4; 6E: quintile 5. The nationwide trend of hospital physicians shown in Fig 6A was most clearly observed in quintile 1 municipalities. In general, the number of physicians younger than 40 years old decreased more as the municipalities became more rural. In the quintile 5 municipalities, the number of physicians younger than 40 years old dropped by a dramatic 59.4%, while the number of those 55–70 and 70 years old and older has increased by 153.0% and 41.0% respectively. The number of physicians younger than 40 years old was the largest of all age groups in 1994, but was the surpassed by older groups and had fallen to third by 2014.

The movement of individual hospital physicians is shown in Table 2 upper part: 1994–2004; lower part: 2004–2014. Physicians were retained less as the municipalities became more rural. Between 1994 and 2004, 77.0% and 36.2% of hospital physicians in the quintile 1 and quintile 5 municipalities were retained in the same quintile respectively. Between 2004 and 2014, the proportion increased to 82.6% and 43.6% respectively, indicating hospital physicians in general has been increasingly less mobile between different quintiles for these 20 years.

**Table 2:** Movement of individual hospital physicians between different quintiles of municipalities (upper: 1994–2004; lower: 2004–2014).

			Municipalities in 2004					
			1 (most urban)	2	3	4	5 (most rural)	Total
Municipalities in 1994	1 (most urban)	n	**47187**	9495	2739	1572	328	61321
		%	**77.0%**	15.5%	4.5%	2.6%	0.5%	100%
	2	n	7922	**24555**	4081	1830	314	38702
		%	20.5%	**63.4%**	10.5%	4.7%	0.8%	100%
	3	n	2779	4261	**10214**	1279	284	18817
		%	14.8%	22.6%	**54.3%**	6.8%	1.5%	100%
	4	n	1283	1745	1097	**3139**	150	7414
		%	17.3%	23.5%	14.8%	**42.3%**	2.0%	100%
	5 (most rural)	n	298	327	268	173	**605**	1671
		%	17.8%	19.6%	16.0%	10.4%	**36.2%**	100%
			Municipalities in 2014					
			1 (most urban)	2	3	4	5 (most rural)	Total
Municipalities in 2004	1 (most urban)	n	**55665**	7789	2681	1059	221	67415
		%	**82.6%**	11.6%	4.0%	1.6%	0.3%	100%
	2	n	8578	**28716**	3881	1397	214	42786
		%	20.0%	**67.1%**	9.1%	3.3%	0.5%	100%
	3	n	2907	4056	**12316**	982	186	20447
		%	14.2%	19.8%	**60.2%**	4.8%	0.9%	100%
	4	n	1161	1600	1188	**3656**	114	7719
		%	15.0%	20.7%	15.4%	**47.4%**	1.5%	100%
	5 (most rural)	n	282	304	257	146	**764**	1753
		%	16.1%	17.3%	14.7%	8.3%	**43.6%**	100%

The age-specific movement of individual hospital physicians who were originally located in quintile 5 municipalities is shown in Table 3 upper part: 1994–2004; lower part: 2004–2014. Between 1994 and 2004, 60.0%, 58.5% and 57.5% of hospital physicians who were 70-, 55–70 and 40–55 years old were retained in the quintile 5 municipalities respectively, while only 23.0% of hospital physicians younger than 40 years old were retained. Between 2004 and 2014, the retention rate of those 70-, 55–70 and 40–55 years old increased to 70.6%, 66.7% and 60.1%, while the rate of those younger than 40 years old fell to 19.3%, suggesting increasing outflow of young physicians from rural areas.

**Table 3:** Age-specific movement of hospital physicians originally located in quintile 5 municipalities (upper: 1994–2004; lower: 2004–2014).

			Municipalities in 2004					
			1 (most urban)	2	3	4	5 (most rural)	Total
Age of physicians in quintile 5 in 1994	-40	n	220	256	188	134	**238**	1036
		%	21.2%	24.7%	18.1%	12.9%	**23.0%**	100%
	40–55	n	52	62	68	29	**286**	497
		%	10.5%	12.5%	13.7%	5.8%	**57.5%**	100%
	55–70	n	24	9	10	8	**72**	123
		%	19.5%	7.3%	8.1%	6.5%	**58.5%**	100%
	70-	n	2	0	2	2	**9**	15
		%	13.3%	0.0%	13.3%	13.3%	**60.0%**	100%
			Municipalities in 2014					
			1 (most urban)	2	3	4	5 (most rural)	Total
Age of physicians in quintile 5 in 2004	-40	n	187	206	138	77	**145**	753
		%	24.8%	27.4%	18.3%	10.2%	**19.3%**	100%
	40–55	n	66	77	96	58	**447**	744
		%	8.9%	10.3%	12.9%	7.8%	**60.1%**	100%
	55–70	n	25	21	19	9	**148**	222
		%	11.3%	9.5%	8.6%	4.1%	**66.7%**	100%
	70-	n	4	0	4	2	**24**	34
		%	11.8%	0.0%	11.8%	5.9%	**70.6%**	100%

## Discussion

The results of this study showed that physicians in Japan’s rural hospitals are older than physicians in urban hospitals and the age gap between rural and urban hospitals has been widening rapidly. The results also showed that, for the past 20 years, the urban-rural disparity in the number of physicians has been widening, which is consistent with the results of recent studies [6–8]. Hospital physicians were less mobile between urban and rural areas than before, whereas the outflow of young hospital physicians from rural to urban areas is substantial and even increasing. Both geographic and age distributions of hospital physicians in Japan have been increasingly disadvantageous for rural areas.

Although the average age of physicians has risen nationwide [24], this study found the trend was most noticeable in the most rural group of municipalities. The marked aging of physicians in rural hospitals can exacerbate the shortage of physicians in rural areas. In the areas with lowest population density (quintile 5), the number of physicians who are not yet 40 years old, the largest group of physicians 20 years ago, has dropped and been surpassed by those 40–55 and those 55–70 years old. This can be a cohort effect, meaning that people who were younger than 40 years old 20 years ago are now between the ages of 40 and 60. But, as shown in Table 3, most of the physicians younger than 40 years old have relocated from rural to urban areas. Most physicians over 55 years old are likely to retire in 15 years, so there is likely to be an even greater shortage of physicians in rural areas in the future.

Japanese workers are at their peak productivity in their 40s [14]. According to a recent large-scale nationwide survey, physicians cut back on their working hours as they grow older. The daytime working hours of physicians in their 60s are on average 80% of physicians in their 20s; the night shift hours of older physicians are 43% of physicians in their 20s [15]. Studies from other countries have also suggested physicians’ clinical performance decreases with age [16,17]. From the perspective of labor force participation, therefore, the aging of physicians in rural hospitals suggests a decline in the physician workforce in rural areas. Moreover, in the Japanese payment system, older physicians earn more than younger ones, which can place a financial burden on rural hospitals. It should be noted, however, older physicians have more clinical experience than younger ones and so have their own value that cannot be measured in terms of working hours.

The new residency training system (training for physicians 1–2 years after graduation) has potentially accelerated the aging of hospital physicians in rural areas [19,20]. Traditionally the personnel of Japanese hospitals depend on physicians pooled in university hospitals. But since the new residency training system was implemented in 2004, university hospitals have lost a substantial amount of newcomers to the pools. This has weakened the physician-assigning power of the universities [25,26]. The effect is more obvious in rural hospitals; than in urban hospitals which have no difficulty attracting young resident physicians. The decrease in the number of hospital physicians less than 40 years old in the most rural group of municipalities (Fig 6E) may reflect this effect.

The new training system for specialist physicians (training for physicians 3–7 years after graduation), scheduled to start nationwide in 2018, can worsen the workforce shortage in small rural hospitals. In this new system, a specialist board certification is given to those who have completed a three- or four-year board-certified specialist program based in large general hospitals, usually university hospitals, unlike the conventional board-certification given based on quality and quantity of cases regardless of training location. Many experts and policy makers have commented that the new training system has the potential to cluster young physicians in urban areas at the expense of rural areas [18–20]. Responding to this concern, the Japanese Medical Specialty Board is encouraging the base hospitals to affiliate with small rural hospitals in their programs and rotate trainees to such hospitals [27]. The Board is also planning to put caps on the total program entrants in five urban prefectures [28]. However, there is no way of knowing if these policies will prevent the concentration of young physicians in urban areas.

Although these changes of physician training system can potentially worsen the physician distribution, some new policies may prevent it from worsening further. For example, since 2008 the Japanese government has dramatically increased the number of entrants to medical schools; most of these entrants were admitted as a *chiikiwaku*, a regional quota of a medical school. The quota had spread from a few to 67 of all the 80 medical schools and the number of entrants is now 1504, accounting for 16% of all medical school students in Japan. Most of the quota entrants are funded by their home prefectures with the understanding that for several years after graduation they will practice in designated areas (mostly rural areas) of the home prefectures [29,30]. The first quota entrants have just started to be assigned to rural areas. The shortage of physicians in rural hospitals may be ameliorated by the expanding number of quota graduates.

The national government is also promoting *chiiki-houkatsu-care*, a community-based system of comprehensive care, in which medical treatment, rehabilitation, long-term care, and preventive services are provided seamlessly within a community [31,32]. This system will shift the role of some physicians from hospital-based inpatient care to clinic-based home-visit care, which may decrease the demand for young hospital physicians in rural areas. At the same time the government is cutting the number of hospital beds. Japan has the largest number of hospital beds per unit population among Organization for Economic Cooperation and Development (OECD) countries [33]. The number varies among prefectures; the prefecture with the most beds has three times as many beds as the prefecture with the fewest [34]. In general, the more rural a prefecture is, the more beds it has. This might be because beds in rural hospitals tend to be used for nursing care instead of medical care. The excessive number of hospital beds has potentially worsened the shortage of physicians in rural areas. By evening out the inter-prefecture variation and by cutting down the total number of beds, the need for hospital physicians in rural areas may be reduced.

As shown in Fig 4C, the average age of clinic physicians decreased between 1994 and 2004, and increased between 2004 and 2014. This is probably due to the skewed age distribution of physicians generally. As Fig 1A and 1B show, in 2004 the number of physicians who were 40–55 years old was the largest among all age groups. This generation of physicians entered medical schools between 1967 and 1982, when the number of medical schools and their entrants doubled [35]. In Japan the age at which hospital physicians become clinic physicians is, by and large, between 40 and 55 years old. These physicians have reached that age in 2004 and probably moved from hospitals to clinics, leading to the decrease in the average age of clinic physicians. Fig 5B, which shows clinic physicians who were 40–55 suddenly increased in 2004, supports this explanation.

The geographically lopsided distribution of physicians is not necessarily problematic. It is worthwhile to concentrate highly specialized medical resources in large cities. High-volume medical facilities with large quantities of specialized human and material resources can provide a better quality of care to patients than low-volume facilities [36–40]. In addition, rather than dispersing resources among many places, channeling them into a few designated facilities can control total health expenditure [41]. This, however, applies to highly specialized care. In terms of care that requires higher accessibility such as primary care and hemodialysis, a geographically equal distribution of care providers contributes to good outcomes [42–46]. Thus policies that assign physicians to geographically appropriate places according to their specialties are quite important. For example, in the new training system for specialist physicians, training programs for subspecialists such as cardiac surgeons who perform highly specialized care should be limited to high-volume hospitals, while programs for general practitioners should follow the distribution of population.

This study has several limitations. The first is that we used the municipality as the geographic unit of analysis. Although the municipality is the smallest and thus the finest unit for analyzing the distribution of physicians, physicians and patients cross municipal borders. Consequently, the municipality-based physician-to-population ratios shown in this study do not necessarily reflect the real demand-supply balance in the areas. In addition, urban and rural physicians have different job responsibilities; specialists are required in urban areas and generalists in rural areas. In this study, no distinction was made between specialists and generalists, so the true demand-supply balance of health care in each area remains unknown. Also there were some non-reporters in the Survey of Physicians, Dentists and Pharmacists. The response rate of the Survey in 2000 was estimated to be 90.4% [47].

The experience of Japan may have a policy implication for other countries. Recruiting and retaining health care providers in rural areas is an important political issue for local and national governments in many countries [48]. It is already known that financial, educational, and professional factors affect where young doctors choose to practice [48–50]. Of note, a study in the United States showed that nearly 30 percent of rural primary care physicians were at or near retirement age, while younger doctors (those under age 40) account for only 20 percent of the current workforce [11,12]. In addition, physicians who spent their career in urban areas were reportedly unlikely to move to rural areas [50–52]. Thus, policies should be considered that encourage physicians (especially those who are young) to choose primary care and recruit them in rural areas. Otherwise, the shortage of rural physicians may only worsen, which will come up as a renewed political issue.

In conclusion, the uneven distribution of physicians is worsening in Japan as is the aging of hospital physicians in rural areas. Policies that prevent the continued maldistribution of physicians and the aging of rural hospital physicians are needed.

## Supporting information

S1 Fig Number of young physicians (under 40 years of age) per 100,000 population in each quintile of municipalities in 1994 and 2014. (TIF)

S2 Fig Proportional change in the number of physicians per 100,000 population in each municipality between 1994 and 2014. (TIF)
