# Supplementary figures and images for: Aging of hospital physicians in rural Japan: A longitudinal study based on national census data
Source: PLoS One. 2018 Jun 1;13(6):e0198317. doi: 10.1371/journal.pone.0198317 (PMC5983464; doi:10.1371/journal.pone.0198317)

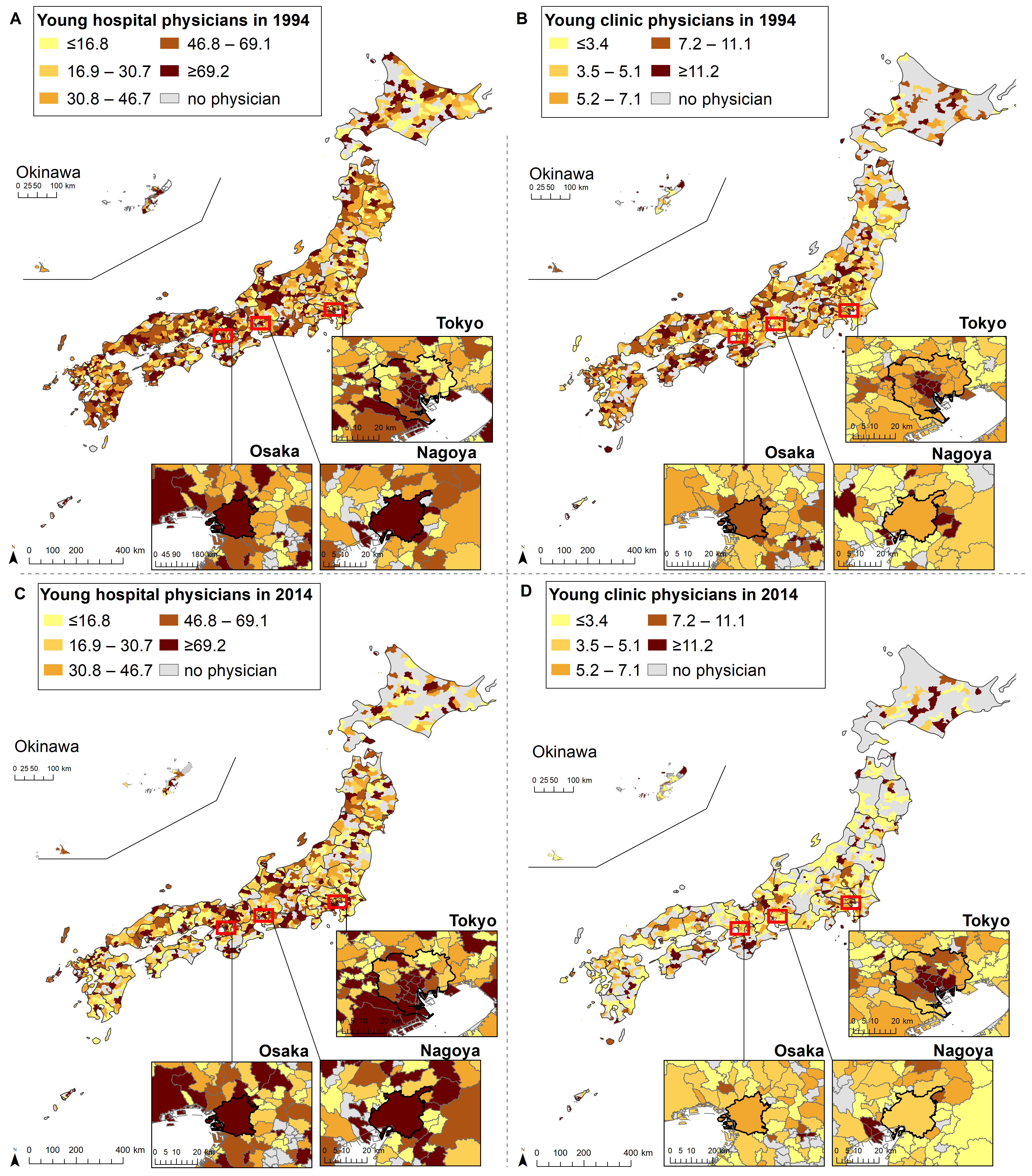

Supplement: S1 Fig — (TIF) [file pone.0198317.s001.tif]

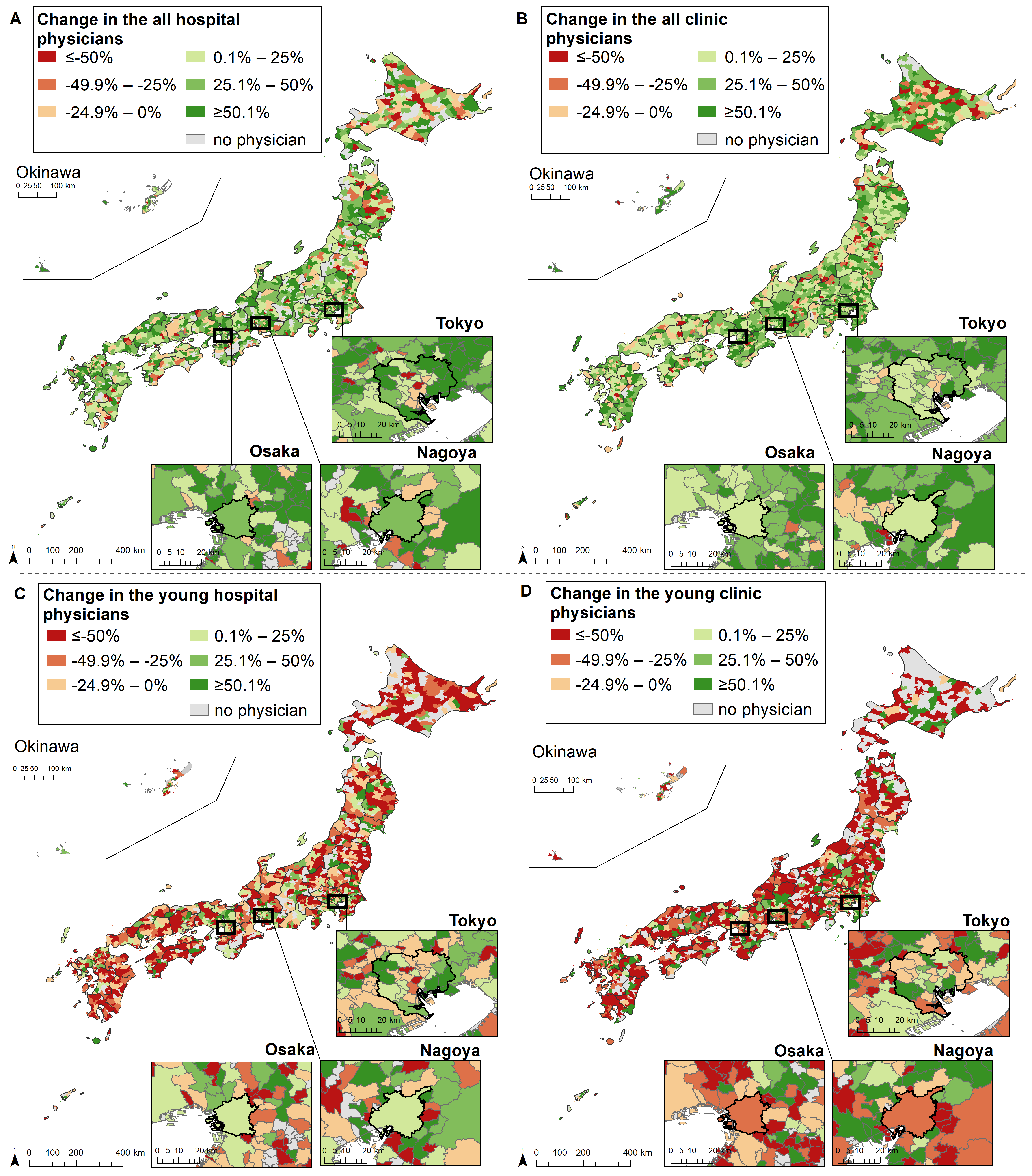

Supplement: S2 Fig — (TIF) [file pone.0198317.s002.tif]
